# Investigation of the atomic layer etching mechanism for Al_2_O_3_ using hexafluoroacetylacetone and H_2_ plasma†

**DOI:** 10.1039/d4tc03615h

**Published:** 2024-11-22

**Authors:** Nicholas J. Chittock, Joost F. W. Maas, Ilker Tezsevin, Marc J. M. Merkx, Harm C. M. Knoops, Wilhelmus M. M. (Erwin) Kessels, Adriaan J. M. Mackus

**Affiliations:** a Department of Applied Physics, Eindhoven University of Technology P.O. Box 513 5600 MB Eindhoven The Netherlands A.J.M.Mackus@tue.nl; b Oxford Instruments Plasma Technology Severn Beach Bristol BS35 4GG UK

## Abstract

Atomic layer etching (ALE) is required to fabricate the complex 3D structures for future integrated circuit scaling. To enable ALE for a wide range of materials, it is important to discover new processes and subsequently understand the underlying mechanisms. This work focuses on an isotropic plasma ALE process based on hexafluoroacetylacetone (Hhfac) doses followed by H_2_ plasma exposure. The ALE process enables accurate control of Al_2_O_3_ film thickness with an etch rate of 0.16 ± 0.02 nm per cycle, and an ALE synergy of 98%. The ALE mechanism is investigated using Fourier transform infrared spectroscopy (FTIR) and density functional theory (DFT) simulations. Different diketone surface bonding configurations are identified on the Al_2_O_3_ surface, suggesting that there is competition between etching and surface inhibition reactions. During the Hhfac dosing, the surface is etched before becoming saturated with monodentate and other hfac species, which forms an etch inhibition layer. H_2_ plasma is subsequently employed to remove the hfac species, cleaning the surface for the next half-cycle. On planar samples no residue of the Hhfac etchant is observed by FTIR after H_2_ plasma exposure. DFT analysis indicates that the chelate configuration of the diketone molecule is the most favorable surface species, which is expected to leave the surface as volatile etch product. However, formation of the other configurations is also energetically favorable, which explains the buildup on an etch inhibiting layer. The ALE process is thus hypothesized to work *via* an etch inhibition and surface cleaning mechanism. It is discussed that such a mechanism enables thickness control on the sub-nm scale, with minimal contamination and low damage.

## Abstract 2

Atomic layer etching (ALE) is required to fabricate the complex 3D structures for future integrated circuit scaling.

## Graphical abstract

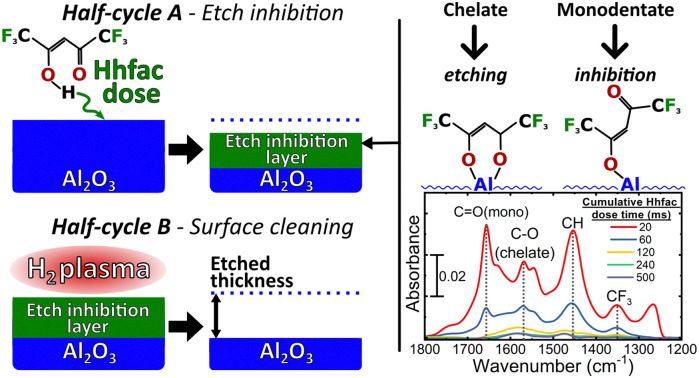

## 1. Introduction

**Fig. 1:**
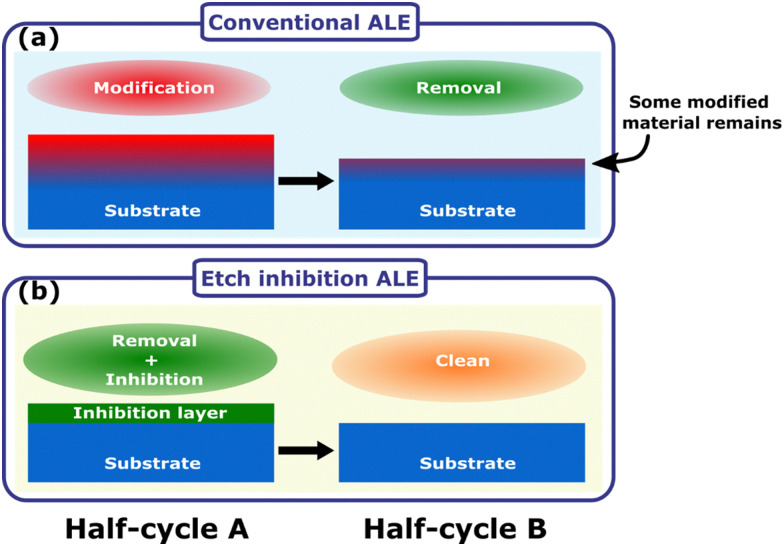
(a) General schematic of an ALE cycle relying on surface modification and ligand exchange, which often leads to a very thin residual contaminated layer at the surface. (b) Etch inhibition and surface clean mechanism, where the etching process self-limits due to formation of an etch inhibition layer. The inhibition layer can then be cleaned away to enable etching in the next cycle.

Isotropic atomic layer etching (ALE) is attracting increasing attention due to its ability to accurately control etch thickness with minimal damage or contamination of the film, qualities which are vital for advanced integrated circuit manufacturing.^1–5^ ALE achieves these requirements through sequentially self-limiting half-cycles, which constitute an ALE cycle.^1–3^ A typical ALE process involves the modification of a thin surface layer in half-cycle A, as shown in Fig. 1(a). This modified layer is then selectively removed in half-cycle B, stopping once the bulk material is reached.^6^ By alternating between these self-limiting steps, the etch depth is accurately controlled by the number of cycles.

Already many ALE processes have been developed based on the approach of surface modification and removal.^7–22^ The most investigated isotropic ALE mechanism is that of fluorination and ligand-exchange, for example for ALE of Al_2_O_3_.^7,17–22^ In such a process, typically hydrogen fluoride (HF) or a F-containing plasma is dosed in half-cycle A until a saturated thickness of metal fluoride is formed at the surface. In half-cycle B a metal–organic etchant molecule, for example trimethylaluminum (TMA, Al(CH_3_)_3_), removes the modified layer. Volatile species are formed through a ligand-exchange reaction, where ligands are transferred from the etchant to the surface. One of the challenges for the use of such a process in device fabrication is contamination of the surface after ALE, either because of incomplete removal of the modified layer leaving behind an oxyfluoride surface, or because the metal etchant molecule introduces undesired metal atoms to the sample surface.^16^ Additionally, high halide exposure can impact stability/performance of reactors, which then requires cleaning, leading to tool down-time.^6,23,24^ As an alternative, some processes based on modification and ligand-addition have been investigated.^25,26^ Rather than dosing a metal–organic molecule as in ligand-exchange, ligand-addition involves dosing the desired ligands directly onto the surface. The mechanism relies on the surface first being modified in half-cycle A, for example by chlorination. In half-cycle B an organic ligand is used to volatilize the modified surface, such as trimethylphosphine (P(CH_3_)_3_) or tetramethylethylenediamine (TMEDA). The mechanism of modification and ligand addition has been shown to be effective for Ni, NiO, CoO, ZnO and Fe_2_O_3_.^25,26^ Although this approach avoids contamination of the etch surface or reactor from a metal–organic etchant, modification of the film is necessary, which may still leave undesired residues.

**Fig. 2:**
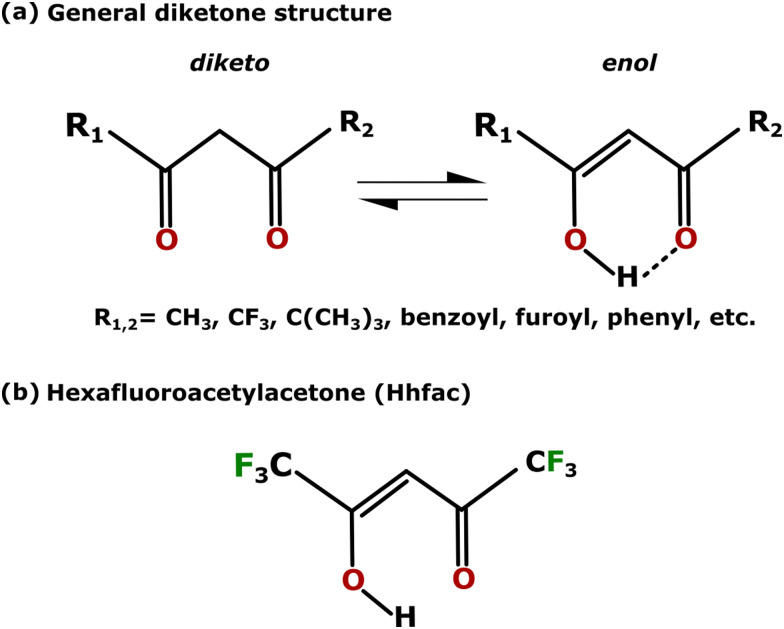
(a) The general structure of a diketone molecule, where R_1_ and R_2_ can be the same or different terminal groups. The molecule is a keto–enol tautomer; in gas phase enol is the more stable configuration for many diketones.^32^ (b) Structure of the hexafluoroacetylacetone (Hhfac) molecule used in this work. Hhfac exists nearly exclusively in the enol form, as shown in the figure.^32^

A group of molecules that is being researched as the etchant for ligand-addition ALE is diketones. The general structure of a diketone molecule is shown in Fig. 2(a). The modification and ligand-addition chemistry with diketones is effective for ALE of metallic films, where typically the first half-cycle is an oxidation or chlorination step to change the oxidation state from the zero state. Once the metal atom is in a higher oxidation state, volatile species can be formed by chelation (*i.e.* forming two coordinate bonds with a metal atom) in the second half-cycle.^8,10,13,27–29^ Furthermore, diketones are tunable molecules, and by adjusting R groups as shown in Fig. 2, the etch rate or selectivity could be tailored in three ways: (i) larger R-groups limit the density of molecules that can bind on the surface due to steric effects.^30^ (ii) More/less electrophilic R-groups affect how strongly bound the H of the hydroxyl group is, adjusting how acidic the molecule behaves.^30–32^ (iii) The volatility of the diketone can be enhanced by increasing intermolecular repulsion between the R-groups, which may promote higher etch rates and lower operating temperatures due to formation of more volatile reaction products.^30,33^ Previously, diketones have been researched as atomic layer deposition (ALD) precursor ligands, as chemical etchants for metal oxide films, and as inhibitor molecules for area-selective ALD (AS-ALD).^7,8,27,33–43^ This large knowledge base can be leveraged to better understand the surface reactions and mechanism for ALE, and to identify materials for which an ALE process will be viable. Previous AS-ALD work has shown that diketones preferentially react with alkaline OH groups on surfaces, and do not readily react with acidic OH groups, potentially helping to predict selectivity of a diketone ALE process.^36,38,44,45^ Additionally, diketones can bind to surfaces in multiple configurations.^44^ For AS-ALD, the different surface bonding configurations were found to play an important role in determining the effectiveness of precursor blocking.^36,44^

Previous work has shown that continuous etching of metal oxides can be achieved by dosing the diketone at high pressure,^40–42^ suggesting that diketones cannot be used for ALE of metal oxides directly. There have, however, been reports in the literature of ALE processes for ZnO and CoO based on diketone etching.^34,46^ The mechanism for metal oxide ALE is hypothesized to follow an etch inhibition and surface cleaning chemistry, as shown in Fig. 1(b). Mameli *et al.* demonstrated that alternating doses of the diketone acetylacetone (Hacac) and O_2_ plasma can isotropically etch ZnO.^34^ Al_2_O_3_ was also found to be etched by this chemistry, resulting in an etch per cycle (EPC) of 0.015 nm at 250 °C.^34^ The ALE mechanism was not investigated in detail, but it was suggested that the Hacac first etches the surface and then forms a carbon-containing layer that needs to be removed by an O_2_ plasma half-cycle. Similarly, Partridge *et al.* showed that CoO ALE is possible using alternating Hacac and ozone (O_3_) exposures. The self-limiting etching behavior was associated with the formation of a carbon layer on the CoO surface, which is then removed by O_3_ exposure. Dosing only Hacac results in a slow etch rate of 0.008 nm per dose, but an EPC of 0.043 nm is achieved when cycling Hacac and O_3_ doses. After O_3_ exposure, the CoO had an oxidized surface of Co_3_O_4_ which is reduced back to CoO by Hacac exposure. The removal of the oxidized surface layer is suggested to contribute to the self-limiting nature.^46^ The controlled ALE behavior reported in these studies is interesting to investigate further as previous metal oxide etching literature suggests continuous etching should occur.^28,40–42^

The goal of this work is to outline an ALE process for metal oxides relying on diketone exposure and surface cleaning. An ALE chemistry of alternating hexafluoroacetylacetone (Hhfac) and H_2_ plasma exposures is investigated.^34^ Hhfac is utilized instead of Hacac due to the lower melting point and higher vapor pressure of Al(hfac)_3_ compared to Al(acac)_3_, which are possible volatile reaction products for each diketone, respectively.^30^ The structure of Hhfac is shown in Fig. 2(b), where the acronym Hhfac denotes the full molecule and hfac the deprotonated form. As previous AS-ALD work has shown that a H_2_ plasma is more effective for the removal of diketone species than an O_2_ plasma,^45^ the suitability of H_2_ plasma for diketone ALE is also tested. Development of this ALE chemistry is performed with the intention to understand the mechanism focusing on two main research questions. Firstly, how does Hhfac adsorb on an Al_2_O_3_ substrate during the ALE process? Secondly, what is the mechanism for self-limiting etching using diketone species on a metal oxide surface? These questions were explored through *in situ* Fourier transform infrared (FTIR) spectroscopy studies and density functional theory (DFT) simulations.

## 2. Method

### 2.1. ALE process development

**Fig. 3:**
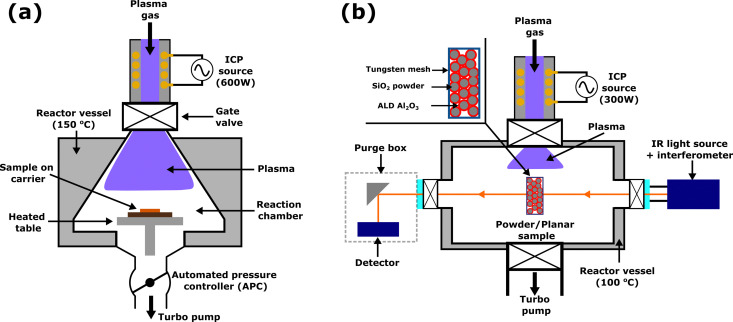
Schematic representations of the reactors used in this work: (a) an Oxford Instruments FlexAL reactor and (b) a home-built ALD reactor equipped with an *in situ* FTIR setup, shown here in transmission mode for powder samples. The angle of the IR mirrors on the detector and source side can be adjusted such that planar samples can be studied in reflection mode. Both systems are pumped with turbo pumps and use an inductively coupled plasma source supplied with 13.56 MHz RF power.

An Oxford Instruments FlexAL reactor with an inductively coupled plasma source, as shown in Fig. 3(a), was used for both ALD and ALE in this work. Al_2_O_3_ samples were grown on 4-inch Si wafers by plasma ALD using trimethylaluminium (TMA) and O_2_ plasma as reactants, with a table temperature of 300 °C and wall temperature of 150 °C. Further details of the reactor and Al_2_O_3_ plasma ALD process can be found in previous reports.^47,48^ ALD was performed for 200 cycles, after which the wafer was diced into approximately 2 × 2 cm^2^ coupons and placed on a 4-inch carrier wafer for ALE processing. Prior to both ALD and ALE processing, samples were heated for 10 minutes while maintaining the chamber pressure at 300 mTorr with Ar gas. After the preheat, all samples were exposed to O_2_ plasma for 2 minutes at 50 mTorr with 200 W ICP power. The ALE process was performed at a table temperature of 350 °C and wall temperature of 150 °C. It is expected that the actual sample temperature is lower than 350 °C due to limited heat transfer from the heated table, through the carrier wafer and to the sample at low pressure. The relationship between the table set point temperature and sample temperature during low-pressure plasma processing has been discussed in more depth elsewhere.^49^ A relatively high table temperature of 350 °C was used to facilitate etching, as previously etching with diketones has been shown to proceed more easily at high temperature.^28,40^ Based on continuous etching studies, 350 °C is near the decomposition temperature of Hhfac,^31,40^ which may be beneficial for the self-limiting behavior as is discussed in more detail later in this work.

**Fig. 4:**
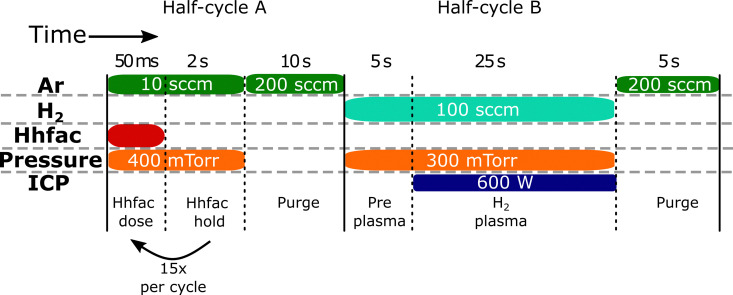
Schematic of the ALE process used in this work. Half-cycle A consists of Hhfac dose/hold steps, which are repeated 15 times per cycle (unless stated otherwise). Half-cycle B is the H_2_ plasma at a pressure of 300 mTorr and 600 W power. Each half-cycle is followed by a 10 s Ar purge with the APC fully open for maximum pumping efficiency.

The ALE process consists of a Hhfac dose/hold half-cycle followed by a H_2_ plasma half-cycle, as shown in Fig. 4, with the chamber continuously pumped by a turbo molecular pump. Plasmas are increasingly being used for isotropic ALE, offering low temperature etch processes, higher etch rates, and the ability to etch materials that are typically more resistant to etching.^16–19,29,34,50–52^ For the plasma step to be isotropic in nature, there should be no substrate biasing during the plasma exposure such that only ions with a low ion energy arrive at the substrate.^16,19^ The H_2_ plasma used in this work for the surface cleaning half-cycle is based on previous AS-ALD literature where the plasma step is used to remove acac ligands from an Al_2_O_3_ surface.^45^ The chamber pressure in the FlexAL is controlled by an automated pressure controller (APC). The angle of the butterfly valve in the APC is automatically adjusted such that the pressure in the chamber is maintained at a set value. Use of an APC enables high chamber pressures with low gas flows, which helps maximize residence time of reactant species.^34,53^ Hhfac (Sigma-Aldrich, 98%) was held in a stainless-steel bubbler at room temperature and then vapor-drawn into the reaction chamber. Hhfac is dosed into the reactor for 50 ms with the chamber pressure set to 400 mTorr. The Hhfac hold is then 2 s with the chamber pressure kept at 400 mTorr before the next 50 ms Hhfac pulse. The hold step is implemented to increase the exposure of the etchant Hhfac to the surface and facilitate the etching reaction. To prevent backflow of the Hhfac into the ICP tube during the hold step, there is a constant 10 sccm Ar flow through the ICP tube. As such, the butterfly valve remains partially open, leading to some of the Hhfac being pumped away during the hold step. Saturation of the Hhfac step is measured as a function of dose/hold pulses rather than as a function of the dose time. After the dose/hold pulses, the reactor is purged for 10 s with an Ar flow of 200 sccm through the ICP tube, with the APC fully open for maximum pumping efficiency. Before the plasma exposure, a 5 s pre-plasma step is included to stabilize the gas flow of 100 sccm H_2_ through the ICP at a pressure of 300 mTorr. The plasma is then struck at an ICP power of 600 W, with the on time varied to determine saturation of the plasma half-cycle. Following the H_2_ plasma, the reactor is purged with 200 sccm Ar with the APC fully open for 5 s.

During ALD and ALE cycles the film thickness was monitored *in situ* using a M-2000 J. A. Woollam spectroscopic ellipsometer (SE) with a photon energy range 1.2–4.9 eV installed at a 70° angle. A Cauchy model was used to fit the Al_2_O_3_ film thickness. After a short exposure of Hhfac an increase in film thickness is observed (∼0.04 nm) which is attributed to adsorbed hfac species. The adsorbed hfac layer was not defined as a separate layer in the model as the optical properties of the adsorbed diketone layer are not known. Atomic force microscopy (AFM) measurements were performed on the Dimension Icon AFM manufactured by Bruker in ScanAsyst mode using a PeakForce-Air tip. The scan frequency was set to 1 Hz and the scan area was 1 × 1 μm^2^.

### 2.2. FTIR studies

Infrared spectroscopy studies were performed on a homebuilt ALD reactor, shown in Fig. 3(b), which is described in detail in ref. 51 and 54. The set-up is equipped with an Invenio S Bruker Fourier transform IR spectrometer with Globar IR source and a liquid N_2_ cooled mercury cadmium telluride (MCT) detector with a spectral range of 12 000–800 cm^−1^. For transmission studies SiO_2_ powder was pressed into a W mesh and heated by passing a DC current through the mesh, with the temperature measured by a thermocouple wire bonded to the mesh. The SiO_2_ powder sample is then coated with Al_2_O_3_ by thermal ALD for 30 cycles, using TMA and H_2_O at 300 °C. Further details on the FTIR set-up and sample preparation can be found in ref. 36. As the powder is directly heated in the mesh, a colder temperature of 300 °C is used for the FTIR ALE study, comparable to the actual sample temperature during process development studies. Hhfac was dosed in static conditions for 20 ms with the gate valves at the top and bottom of the reactor vessel closed. Hhfac was then held in the reactor for 2 s before opening the gate valve to the pump. The top gate valve is then opened, and the chamber is purged with Ar for 10 s before an FTIR measurement was taken. H_2_ gas flow was controlled *via* a needle valve to give a chamber pressure of 100 mTorr with both gate valves open. After 5 s of gas stabilization, the H_2_ plasma was struck using an ICP power of 300 W. A lower ICP power is utilized on the homebuilt system, due to differences between the homebuilt system and the FlexAL system. The chamber is then purged for 10 s with Ar. During an FTIR measurement the chamber was pumped without gas flow. Spectra presented in this work are difference spectra referenced either to a common starting surface, or to the previous measurement to investigate changes in surface bound species. In FTIR analysis of a surface, the presence of a positive peak in the spectrum indicates the addition of species to the surface, while a negative peak denotes removal of species from the surface.

Planar samples were also employed for FTIR studies, utilizing the FTIR set-up on the homebuilt system in reflection mode. Samples were prepared on a planar Al substrate using the same thermal Al_2_O_3_ ALD process as for the powder samples. Here the sample is placed directly onto the substrate table, which is heated to 300 °C. A lower temperature is employed as the heat is transferred directly from the table to the sample, instead of through a carrier wafer.^49^

### 2.3. Computational methods

The Vienna *ab initio* Simulation Package (VASP, version 5.4.4) was used to perform all density functional theory (DFT) calculations reported in this study.^55–57^ The projector augmented wave (PAW) method was employed to describe electron–ion interactions.^58,59^ Calculations were performed based on Perdew–Burke–Ernzerhof (PBE) exchange–correlation functional of the generalized gradient approximation (GGA), and the influence of the van der Waals interactions were considered by the dispersion correction D3 and the Becke–Johnson (BJ) damping function.^60–62^ A kinetic energy cutoff of 400 eV was used for the plane-wave basis set. The convergence criteria for structural optimizations were set such that the total forces acting on each atom must be smaller than 0.01 eV Å^−1^. Convergence criteria for the self-consistent-field cycle was set to 10^−5^ eV. Gaussian smearing of 0.01 eV was used throughout the study. The Brillouin zone of crystalline Al_2_O_3_ bulk was integrated using an automatically generated *Γ* centered 11 × 11 × 5 *k*-point mesh, whereas a *Γ* centered 2 × 2 × 1 Monkhorst–Pack grid was used for the surface calculations.^63^ A 4-layer 3 × 3 supercell of α-Al_2_O_3_(0001) was modeled following the same procedure as our previous works.^36,45,64^ Periodicity of the slab in the direction perpendicular to the surface was avoided by adding a vacuum spacing of 15 Å. The resultant supercell has the *a*, *b*, *c* lattice dimensions of 14.21 Å, 14.21 Å, and 26 Å, respectively with *θ* = 120°. To achieve a hydroxylated Al_2_O_3_ (0001) surface, the Al_2_O_3_ surface is partially hydroxylated by locating hydroxyl groups on all 9 top layer Al atoms, resulting in a 1 monolayer OH coverage (corresponding to 5.1 OH nm^−2^). This is slightly less than the experimentally observed value of 7.1 OH nm^−2^,^65^ which is due to the difference between an amorphous surface and the perfectly crystalline surface employed for simulations. In order to balance the negative charge from the addition of OH, 9 out of the 18 exposed O atoms neighboring the top layer Al atoms are hydrogenated. The bottom two Al_2_O_3_ layers were kept frozen at their bulk positions. More than 20 different starting configurations were studied for the Hhfac adsorption of which the resulting unique configurations are reported here. The Hhfac adsorption energies were calculated usingΔ*E*_ads_ = *E*_slab+Hhfac_− (*E*_slab_ + *E*_Hhfac_).where *E*_slab+Hhfac_ is the total energy of the (hydroxylated) Al_2_O_3_ (0001) slab with adsorbed Hhfac at its lowest energy position on the surface, *E*_slab_ is the total energy of a clean slab, and *E*_Hhfac_ is the energy of an isolated Hhfac molecule. The energy of the isolated Hhfac molecule was computed *via* spin relaxed calculations in 20 Å cubic cells at the gamma point. The inputs for the adsorption geometries were prepared using Maestro Materials Science 5.0.122, Release 2023-2, whereas all visual representations of the computed geometries were prepared using Vesta v.3.5.7.^66,67^

## 3. Results

### 3.1. ALE process development

**Fig. 5:**
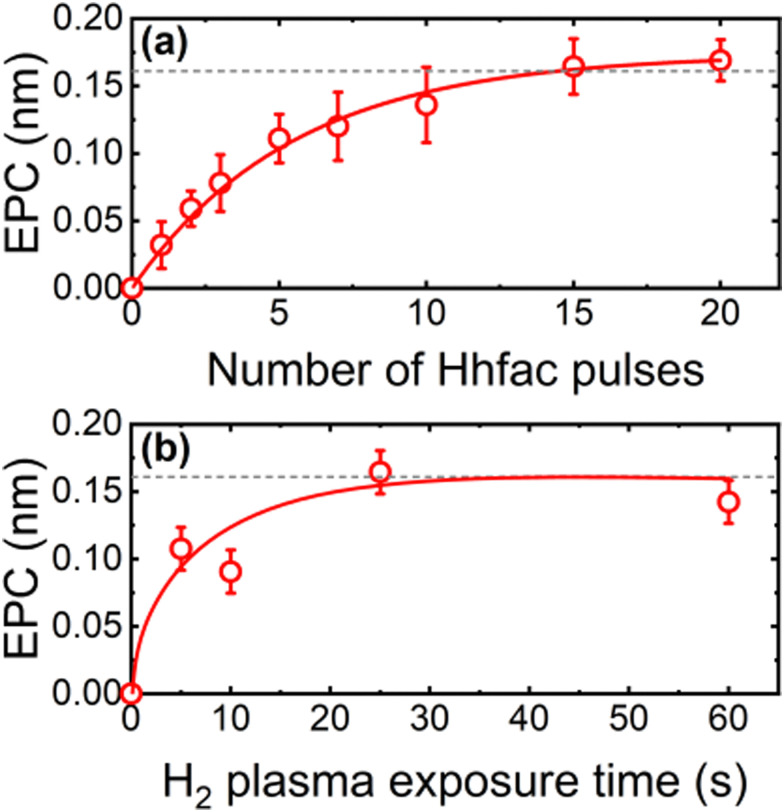
Saturation curves at 350 °C for (a) Hhfac pulses using a 25 s H_2_ plasma and (b) H_2_ plasma exposure using 15 × 50 ms pulses of Hhfac per cycle. The lines are guides to the eye.

The self-limiting nature of the half-cycles is investigated on planar samples using *in situ* SE to assess whether controlled etching can be achieved. Saturation curves for both half-cycles at 350 °C table temperature can be seen in Fig. 5. For each data point, 25 cycles of ALE were performed, and the film thickness was measured every 5 cycles. Hhfac saturation is shown in Fig. 5(a) as a function of the number of 50 ms Hhfac pulses per cycle, while using a 25 s H_2_ plasma in half-cycle B. Initially, the EPC increases quickly and then appears to saturate after 15 pulses per cycle. Slight soft-saturation of the Hhfac step may be occurring as is discussed in Section 3.2. Similar soft-saturation behavior has been observed previously for etching with diketones.^41,46^ The H_2_ plasma saturation curve (for 15 × 50 ms pulses of Hhfac per cycle) in Fig. 5(b) shows that saturation occurs at 25 s H_2_ plasma. The combination of soft-saturation for the Hhfac half-cycle and saturation for the H_2_ plasma half-cycle gives controlled etching of Al_2_O_3_ with an EPC of 0.16 ± 0.02 nm at 350 °C.

**Fig. 6:**
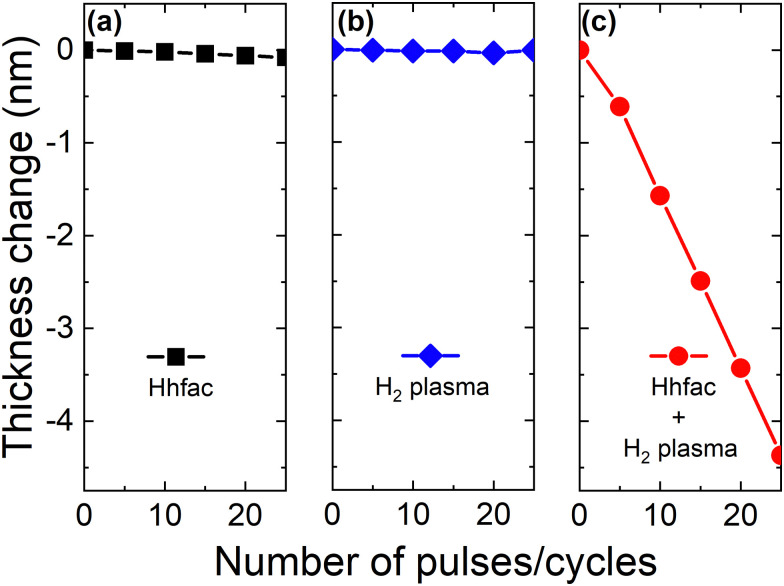
Thickness evolution as a function of pulses/cycles for (a) only Hhfac dosing, (b) only H_2_ plasma exposure and (c) ALD cycles with Hhfac and H_2_ plasma pulses. Experiments were performed at 350 °C table temperature.

To confirm the controlled nature of this ALE process, a synergy test is performed, as shown in Fig. 6. Synergy is an important parameter for ALE, demonstrating that etching should only occur when alternating between the two reactants.^1^ The synergy parameter, *S*, is defined as
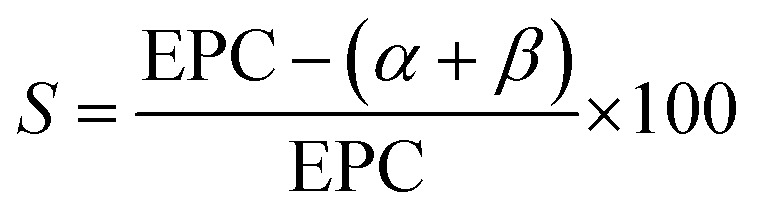
where EPC is the ALE etch per cycle, and *α* and *β* the continuous etch components from half-cycle A and B, respectively.^1^Fig. 6(a) shows the change in film thickness when only exposing the substrate to Hhfac half-cycles (half-cycle A). Over the 25 steps there is a small thickness decrease of 0.08 nm, corresponding to an *α* value of 0.003 ± 0.001 nm per step. This small continuous etch component is shown more clearly in Fig. S1 (ESI†), and can explain why there is soft-saturation of the Hhfac exposure in Fig. 5(a). Additionally, a small increase (0.04 nm) in film thickness is observed after the first Hhfac pulse which is due to adsorption of Hhfac on the surface. Repeated exposure to the H_2_ plasma step, shown in Fig. 6(b), gives a negligible thickness change after 25 pulses of H_2_ plasma, yielding a *β* value of 0.001 ± 0.001 nm per step. Fig. 6(c) shows the change in thickness when performing full ALE cycles. A linear fit through the points gives an EPC value of 0.17 ± 0.01 nm, in agreement with the saturation curves in Fig. 5. Using the etch rates determined from Fig. 6(a)–(c), a synergy value of 98% is calculated. The high synergy value highlights that this ALE process is near ideal, with minimal continuous etching components.

Furthermore, the effect of table temperature was investigated as reported in Fig. S2 (ESI†), demonstrating that high process temperatures result in a higher EPC. For table temperatures of 250 °C and 300 °C the EPC is 0.04 ± 0.01 nm and 0.08 ± 0.01 nm, respectively. Similar temperature-dependent etch behavior has been observed using diketones for continuous etching,^40^ ALE of metals,^7,8,10^ and metal oxide ALE.^34,46^ Interestingly, the EPC for the Hhfac ALE process at 250 °C (0.04 ± 0.01 nm) is higher than the 0.015 nm reported for a similar Hacac/O_2_ plasma process at the same temperature.^34^ This higher EPC could be attributed to some combination of different aspects such as enhanced surface cleaning by the H_2_ plasma, higher volatility of the etch product, or *in situ* F radical production from dissociation of hfac ligands. More effective surface cleaning by H_2_ plasma compared to O_2_ plasma has been observed in AS-ALD.^45^ Enhanced removal of the surface inhibiting layer reduces steric hinderance during the next Hhfac dose, thus facilitating etching.^45^ Additionally, the higher volatility of the proposed etch product (Al(hfac)_3_ compared to Al(acac)_3_) may enable the etch product to leave the surface more easily, allowing for more etching to occur before surface inhibition.^30,33^ Potential fluorination of the surface to AlF_*x*_ can increase the etch rate as will be discussed further in Section 3.4.

**Fig. 7:**
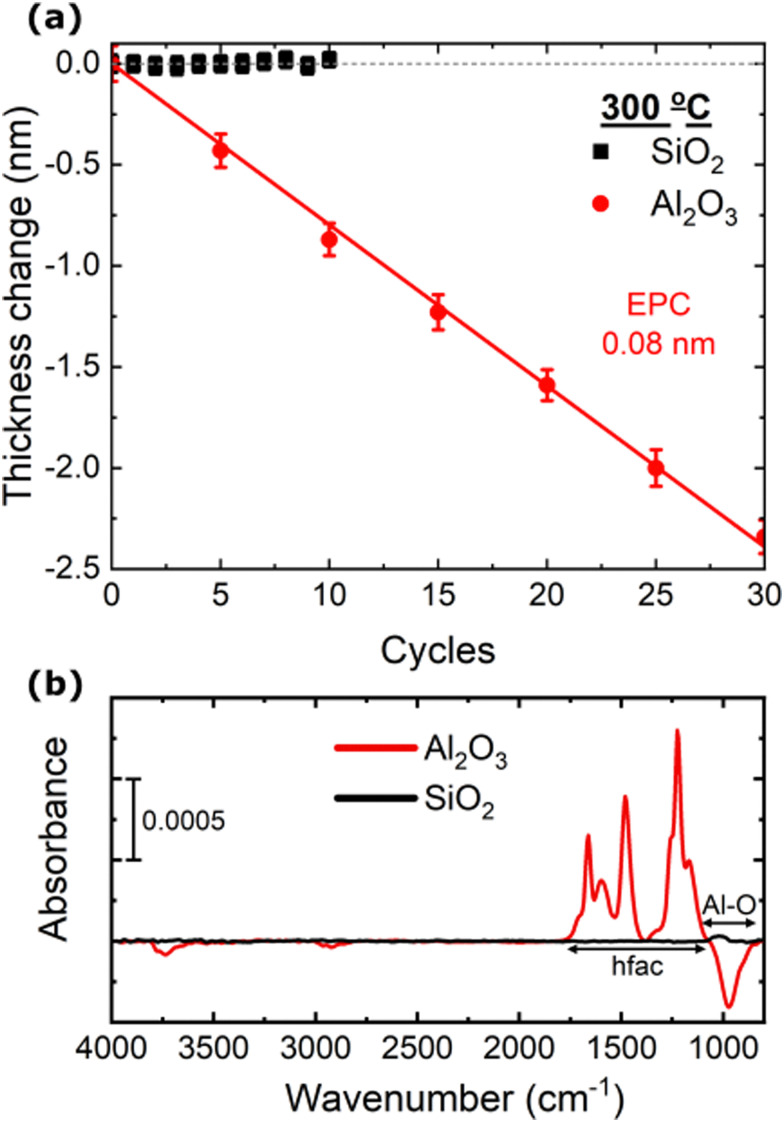
(a) Etched thickness as a function of ALE cycles using 15 pulses of Hhfac dose/hold and 25 s H_2_ plasma each cycle. Experiments were performed at 300 °C table temperature, yielding an EPC of 0.08 ± 0.01 nm per cycle. (b) Reflection mode absorbance spectra for Al_2_O_3_ and SiO_2_ planar films exposed to a 100 ms Hhfac dose, referenced to their as-deposited surfaces. The Al–O and hfac absorbance regions are highlighted in the figure.

Important characteristics of an ALE process are the abilities to smooth surfaces and to etch selectively. These two qualities are often required for integration into device fabrication flows.^1–6^ The roughness of the Al_2_O_3_ film was observed to decrease with the etched thickness. After 3 nm of ALE the RMS roughness decreased from 0.50 ± 0.07 nm to 0.24 ± 0.07 nm, with the roughness value approaching that of the Si substrate underneath (0.20 ± 0.05 nm), as shown in Fig. S3 (ESI†).^1,2,68^ Selectivity is desired such that the ALE process does not etch masks or other functional layers of the device. The selectivity of the Hhfac/H_2_ plasma ALE chemistry for etching Al_2_O_3_ with respect to SiO_2_ was tested at 300 °C. Fig. 7(a) shows the etched thickness as a function of ALE cycles for SiO_2_ and Al_2_O_3_ films exposed to the Hhfac/H_2_ plasma ALE process. There is negligible change in the SiO_2_ film thickness after 10 ALE cycles, highlighting that the Hhfac/H_2_ plasma ALE process allows for near perfect selectivity between SiO_2_ and Al_2_O_3_. The high selectivity is attributed to a lack of Hhfac adsorption on the SiO_2_ surface.

To support our hypothesis for selective etching, FTIR spectra for SiO_2_ and Al_2_O_3_ films after exposure to Hhfac are compared. Both films are exposed to a 100 ms Hhfac dose and referenced to their respective as-deposited surface, as shown in Fig. 7(b). The graph highlights the selective adsorption: Al_2_O_3_ has peaks indicating hfac adsorption, as well as a reduction in absorbance around the Al–O bond region. In contrast, on the SiO_2_ film no change is observed after the Hhfac exposure. For the Al_2_O_3_ surface there is also a decreased absorbance at 2940 cm^−1^ and 3700 cm^−1^, which are attributed to the removal of CH_3_ and OH groups, respectively. The CH_3_ groups are likely coming from unreacted ligands from the ALD process that are removed by the H_2_ plasma half-cycle, while OH groups are consumed due to the diketone reaction with the surface, as is discussed in Sections 3.2 & 3.3. There is no change in the spectrum in these regions for the SiO_2_ surface, which is further evidence that the diketone Hhfac is not reacting with the surface. The selectivity observed here for Hhfac is similar to results from AS-ALD studies, where the diketone Hacac was shown not to adsorb on an acidic SiO_2_ surface, while strongly bonding to more alkaline surfaces like Al_2_O_3_.^36,38,44^ The spectra in Fig. 7(b) support the hypothesis that the selective etching in this work is enabled by a lack of Hhfac adsorption on SiO_2_. Furthermore, these observations suggest that a diketone-based ALE chemistry would not etch materials with acidic OH groups, such as GeO_2_ and WO_*x*_, while selectively etching materials with alkaline OH groups like CoO_*x*_ and CuO_*x*_.^38,43^

**Fig. 8:**
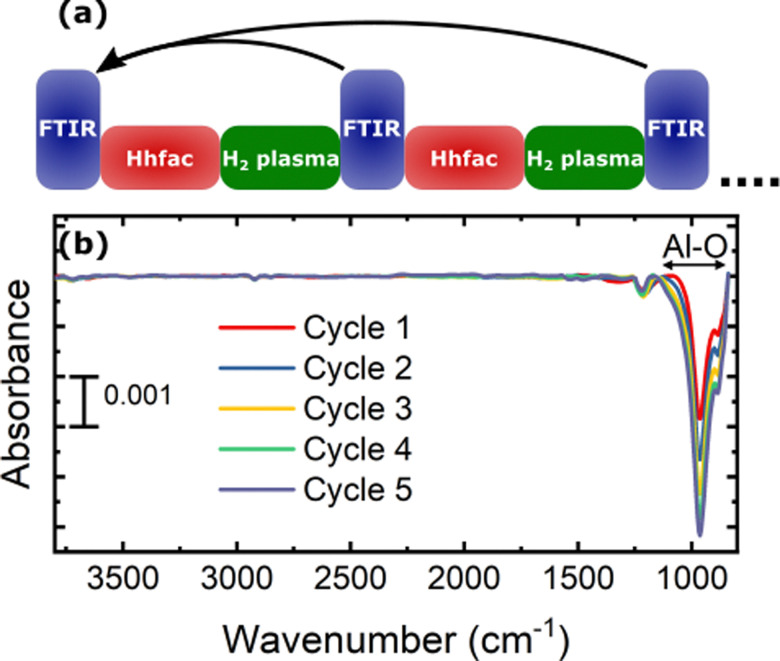
(a) Schematic of the performed experiment to determine whether there is any build-up of Hhfac residue on the Al_2_O_3_ surface post ALE. FTIR spectra are referenced to the spectrum for the as-deposited Al_2_O_3_ planar film. (b) Spectra taking in reflection mode after full ALE cycles. A cyclic decrease in absorbance for the Al–O bond region is observed. For the rest, the spectrum shows minimal change over the 5 ALE cycles.

Reduced surface contamination post etching is an anticipated benefit of ALE compared to conventional etch techniques.^1,2^ The potential surface contamination from the ALE process is investigated by performing FTIR measurements on planar samples. An FTIR spectrum was taken after each full ALE cycle, with the spectrum of the as-deposited Al_2_O_3_ acting as the reference as illustrated in Fig. 8(a). The spectra taken over the 5 ALE cycles shows evidence of etching as the Al–O bond absorbance decreased with each cycle. The lack of peaks in the spectra between 1900–1200 cm^−1^, which are indicative of hfac ligands, demonstrates that the 25 s H_2_ plasma is sufficient for the removal of hfac species from a planar sample. As the only change in the absorbance spectrum over the ALE cycles is removal of Al–O bonds, it can be concluded from Fig. 8 that the ALE process leads to minimal contamination of the film.

### 3.2. Mechanism studies

While the process enables the controlled etching of Al_2_O_3_, there is an open question of what the self-limitation mechanism is for the Hhfac half-reaction. To study the ALE mechanism in detail, FTIR studies were performed looking at both the Hhfac and H_2_ plasma exposures at 300 °C.

**Fig. 9:**
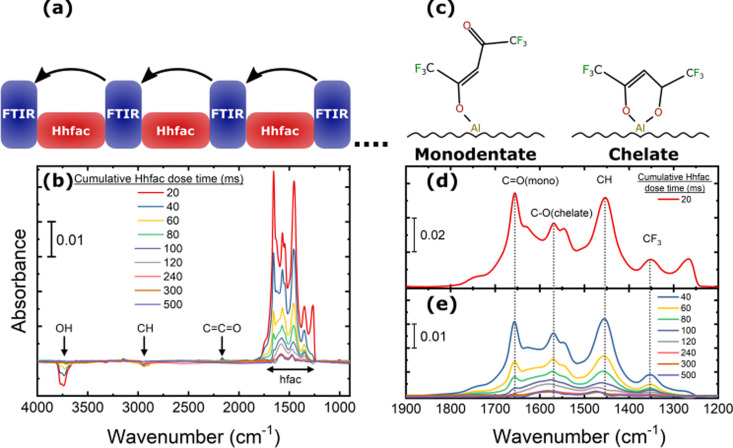
FTIR difference spectrum for adsorption of Hhfac on the Al_2_O_3_-coated powder. (a) Illustration of the performed experiment. The arrows indicate the reference spectrum for each measurement. (b) The spectra between 4000–900 cm^−1^ for different dosing times, listing the peaks discussed in the main text. (c) Schematic of the monodentate and chelate bonding configurations of hfac on the surface. (d), (e) Zoom in on the region 1900–1200 cm^−1^ from panel (a) for (d) the first 20 ms Hhfac pulse and (e) for increasing cumulative Hhfac dose times. In (d) and (e) the main monodentate and chelate peaks are highlighted in the figure.

To monitor how the Hhfac binds to the surface during ALE, FTIR measurements were taken for sequential doses of Hhfac as illustrated in Fig. 9(a). The difference spectra for increasing total Hhfac dose times are shown in Fig. 9(b). A decrease in absorbance is observed at 3700 cm^−1^, which is attributed to OH consumption during the initial Hhfac adsorption. As Hhfac adsorbs, it is expected that an H atom is transferred from the Hhfac molecule to the surface which may then react with a surface OH group to form volatile H_2_O. The absorbance in the OH region is not observed to change after 80 ms of Hhfac dosing. There is also a small negative peak at 2940 cm^−1^ attributed to C–H bonds, which could indicate removal of surface carbon species. At 2163 cm^−1^ a small positive peak from a surface ketene structure (C=C=O) is present, perhaps due to decomposition of the hfac species.^31,69^ Previously, Hhfac has been shown to decompose and form similar ketene structures on ZnO powder at temperatures of 427 °C (700 K).^31^

Between 1900–1200 cm^−1^ there is a significant increase in absorbance for the first Hhfac dose, which can be seen in more detail in Fig. 9(d). This absorbance increase suggests many hfac molecules are adsorbed onto the surface in the first pulse. Based on simulated bond vibrational frequencies (Table S1, ESI†) and literature studies, the contributions of the different diketone bonding configurations were determined.^36^ The largest hfac peak at 1656 cm^−1^ is attributed to hfac bound in monodentate configuration. In contrast, for AS-ALD studies utilizing the diketone Hacac at 150 °C the dominant bonding configuration is observed to be the chelate configuration.^36^ The different surface bonding configurations are shown in Fig. 9(c). For Hhfac adsorption in this study there is a relatively small peak at 1571 cm^−1^ related to the chelate configuration after the first 20 ms pulse. Difference spectra in Fig. 9(e) show that each subsequent Hhfac pulse results in a smaller increase in absorbance between 1900–1200 cm^−1^, suggesting each pulse is adding fewer hfac species to the surface. This behavior indicates that the surface is becoming saturated, and that the reaction of Hhfac with the surface is self-limiting. The small change observed after 500 ms total dose time could be due to reorganization of hfac ligands on the Al_2_O_3_ surface. Such reorganization may enable the small continuous etch component observed for planar samples (Fig. 6(a)).

**Fig. 10:**
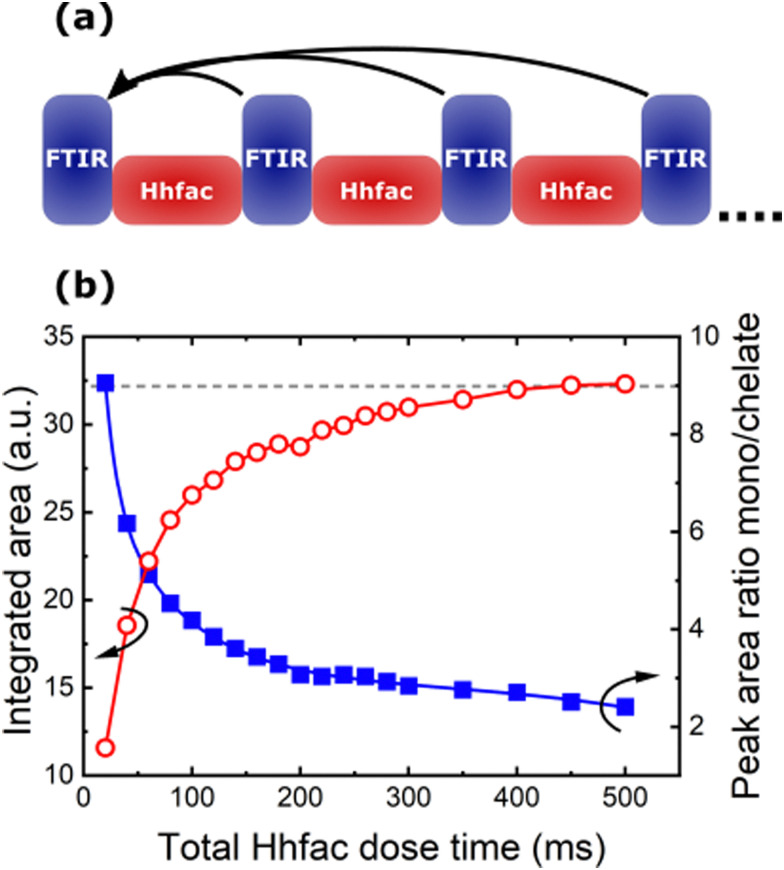
(a) Representation of the procedure performed to analyze the integrated total area in the region 1900–1200 cm^−1^. The data is the same as Fig. 9, however all the FTIR spectra are reference to the starting surface, such that the total change in the surface can be measured. (b) Integrated absorbance area between 1900–1200 cm^−1^ (red, open circles), and the peak area ratio of monodentate (1656 cm^−1^) to chelate (1571 cm^−1^) hfac (blue, closed squares) as a function of Hhfac dose time.

The saturation behavior can also be seen by tracking the integrated area of the spectra between 1900–1200 cm^−1^ as a function of Hhfac dose time. For these measurements the spectra are referenced to the starting surface as shown in Fig. 10(a). The full spectra used as a reference can be seen in Fig. S4 (ESI†). Initially there is a large increase in the integrated area, suggesting that many hfac ligands are being adsorbed onto the surface. As Hhfac dose time increases, the increase in integrated area after each Hhfac pulse is less than the one before. The increase in integrated area between 450 ms and 500 ms Hhfac dosing is minimal, as can be seen in Fig. 10(b), which indicates that the surface has become saturated with hfac ligands. Determination of the peak area at 1656 cm^−1^ and 1571 cm^−1^ allows for comparison of the monodentate and chelate bonding configurations as a function of total Hhfac dose time. The monodentate peak is initially significantly larger than the chelate peak, suggesting that there is a larger amount of monodentate species on the surface after 20 ms. As the total Hhfac dose time increases, the relative amount of monodentate and chelate on the surface changes, with relatively more chelate present as the total dose time increases. Eventually the hfac coverage starts to saturate, with the monodentate configuration remaining to be the dominant surface species once saturation is reached.

**Fig. 11:**
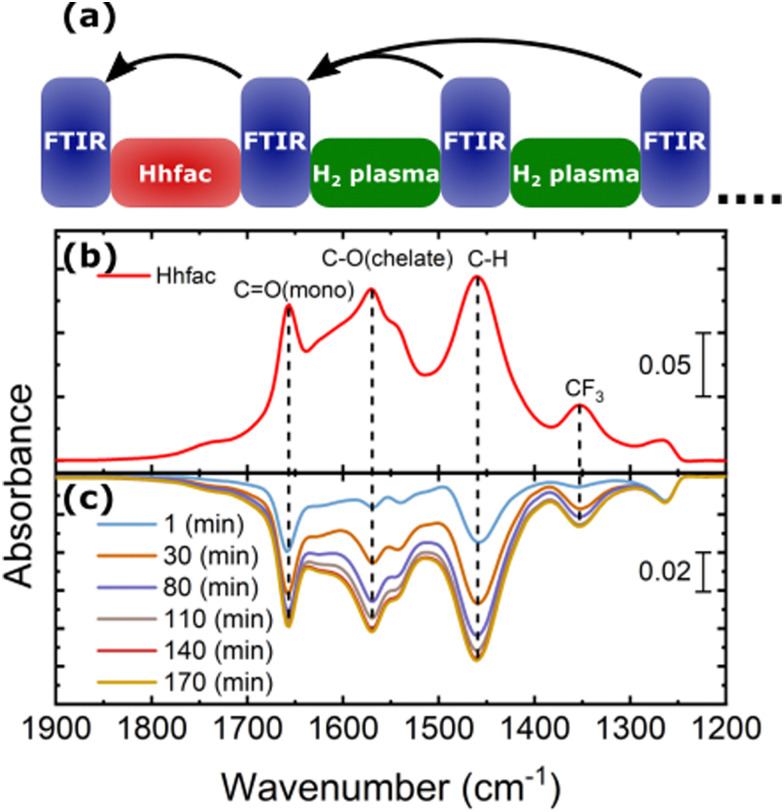
(a) Schematic of the performed experiment investigating the effectiveness of H_2_ plasma at removing hfac ligands adsorbed on the Al_2_O_3_ surface. The surface is first functionalized by carrying out a 500 ms Hhfac exposure. The arrows indicate the reference spectrum that is used for each measurement. (b) Spectrum for Al_2_O_3_ exposed to 500 ms Hhfac dosing, which is shown such that a comparison to the removal spectra can easily be made. (c) The difference spectra for increasing H_2_ plasma exposure times referenced to the spectrum in (b).

For the ALE process to continue, the hfac surface species must be removed by a surface cleaning step, which in this work is performed by H_2_ plasma. Spectra for increasing H_2_ plasma exposure time are shown in Fig. 11(b), in which a negative absorbance indicates removal of species from the surface. After 1 minute of H_2_ plasma, there is a decrease in the absorbance spectra in the 1900–1200 cm^−1^ wavenumber range, which is due to the removal of hfac ligands from the surface. A decrease in absorbance at 2163 cm^−1^ is also observed, indicating that the ketene structures are removed. No other significant changes were observed in the spectra recorded during the H_2_ plasma exposure.

As the H_2_ plasma exposure time increases there is continued removal of the hfac species from the Al_2_O_3_ surface, in agreement with observations from AS-ALD studies.^45^ Removal of the hfac species from the Al_2_O_3_-coated powder is observed to saturate after 140 minutes of H_2_ plasma exposure, which is significantly longer than the 25 s used on planar samples. As shown in Fig. 8, a 25 s H_2_ plasma left no residue on a planar sample. The long plasma exposure time required for the high-surface-area powder sample can be explained by the difference in penetration depth of the Hhfac molecules and H radicals. The Hhfac molecules penetrate deeper, while the H radicals recombine at the surface of the powder. The smaller negative absorbance feature for the H_2_ plasma removal spectra as compared to the positive peaks from Hhfac adsorption suggests that some hfac species have remained on the powder sample, likely beyond the penetration depth of the H radicals.

As some applications may not be compatible with H_2_ plasma exposure, alternative plasma chemistries for the surface cleaning half-cycle should be explored. Previous AS-ALD literature suggests that O_2_ plasma can be used for surface cleaning, however it is not as effective as H_2_ plasma in completely removing ligands bound to the surface.^45^ Additionally, isotropic ALE of ZnO using alternating exposures of Hacac and O_2_ plasma has been demonstrated.^34^ Thus, it is expected that O_2_ plasma could be a viable alternative for H_2_ plasma. Removal of hfac ligands from an Al_2_O_3_ surface with O_2_ plasma is tested in the work, as shown in Fig. S5 (ESI†). The FTIR data indicates that O_2_ plasma can remove the hfac ligands from the Al_2_O_3_ surface and would therefore likely be a viable alternative reactant to H_2_ plasma.

### 3.3. Simulation results on Hhfac adsorption

**Fig. 12:**
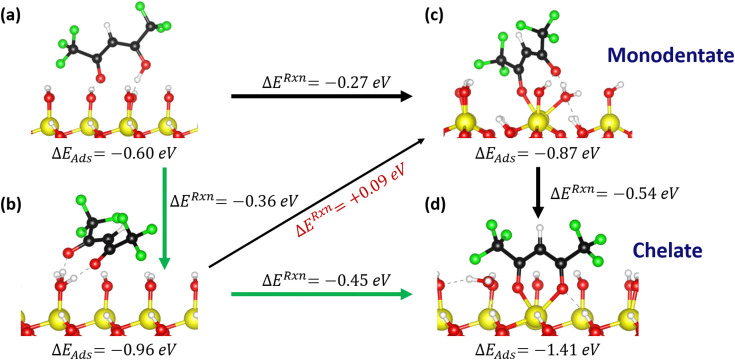
Adsorption pathway for Hhfac on a hydroxylated Al_2_O_3_ surface. (a) Hhfac molecule initially physisorbs to the surface. (b) Donation of H from Hhfac to the surface, forming H_2_O on the surface. (c) The hfac ligand bound in monodentate configuration, with the H_2_O still bound to the surface. (d) Chelate configuration of the hfac ligand, with both O atoms bound to the same surface Al atom. Adsorption energies for each configuration are shown beneath the images. The arrows indicate potential transitions on the surface and the difference in energy between two configurations.

**Fig. 13:**
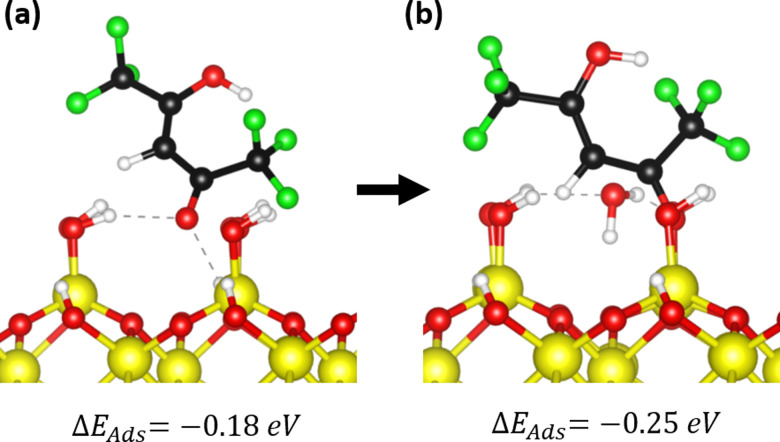
(a) Physisorption of a Hhfac molecule onto an OH-terminated Al_2_O_3_ surface where only one O atom is directed towards the surface. From configuration (a) the Hhfac can transition into (b) a monodentate bound configuration with an OH group directed away from the surface.

DFT studies were employed to understand the surface reactions and bonding configurations of hfac on Al_2_O_3_ in more detail. Considering an OH-functionalized Al_2_O_3_ surface, it was observed that physisorption of the Hhfac is energetically favorable, agreeing with the experimental observation that Hhfac adsorbs on an Al_2_O_3_ surface. Physisorption can occur with both O atoms pointing toward the surface as in Fig. 12(a), or with the C-chain twisted and only one O pointing towards the surface, Fig. 13(a). Both routes are energetically favorable, however the configuration in Fig. 12(a) is more favorable (Δ*E*_ads_ = −0.60 eV *vs.* Δ*E*_ads_ = −0.18 eV).

Considering the more favorable physisorption configuration in Fig. 12(a), there are two possible pathways which both lead to the H from the Hhfac being transferred to the surface, generating H_2_O as shown in Fig. 12(b) and 12(c). The molecule can directly bind in the monodentate configuration (Δ*E*_ads_ = −0.87 eV) as shown in Fig. 12(c), or it can take a more energetically favorable configuration (Δ*E*_ads_ = −0.96 eV) with both O atoms pointing at the surface as in Fig. 12(b). In the lower energy configuration shown in Fig. 12(b), the surface bound H_2_O helps to stabilize the de-protonated hfac through hydrogen bonds.^44^ From the configuration in Fig. 12(b) the hfac molecule can bind to a surface Al atom, either by transitioning through a monodentate configuration (Fig. 12(c)) with a minimum thermodynamic barrier of 0.09 eV, or directly to the chelate configuration (exothermic, Δ*E* = −0.45 eV) shown in Fig. 12(d). The monodentate configuration may thereby act as a transition state with an activation barrier of Δ*E* = 0.09 eV towards forming the chelate bound species.

The energetically favorable conversion from the physisorbed to the chelate configuration suggests that if there is space on the surface, the hfac ligands will bind in the chelate configuration. The DFT analysis however does not consider potential steric hinderance of neighboring hfac surface species or detailed investigation of the energy barriers, which would require nudged elastic band analysis. It is interesting that experimentally the dominant configuration in the FTIR spectrum during the initial doses is monodentate bound hfac, as simulations suggest that chelate is more favorable. The implication of these observations are discussed in Section 3.4.

The pathway shown in Fig. 12 can lead to the formation of chelate bound species, which are hypothesized to be important for the etching reaction in the first half-cycle.^34^ However, to enable the controlled nature of ALE the etching reaction must also eventually self-limit. Thus, it is interesting to look at potential pathways for generating other hfac bonding configurations during the Hhfac half-cycle. Considering Hhfac adsorption where only one of the O atoms is pointing towards the surface, a less energetically favorable physisorption configuration is found, as shown in Fig. 13(a). The molecule can transition from the physisorbed to an adsorbed configuration (exothermic, Δ*E* = −0.07 eV) by transferring a H atom to a surface OH group and forming H_2_O, as can be seen in Fig. 13(b). From the monodentate configuration in Fig. 13(b) it is unlikely that the molecule will transition into a chelate configuration, as it would require a 180° rotation of the C chain. Rotation of the C chain in the gas-phase has an energy barrier of >1 eV, as shown in Fig. S6 (ESI†), and is therefore unlikely to occur when the molecule is adsorbed on the surface. The monodentate configuration shown in Fig. 13(b) is expected to remain in this configuration and contribute solely to surface inhibition. By not having a viable pathway to the chelate configuration, the configuration in Fig. 13(b) may facilitate the formation of an etch-inhibition layer on the Al_2_O_3_ surface, and thus enable the self-limiting behavior.

### 3.4. Discussion

The main goals of this work were to determine if Hhfac and H_2_ plasma can be used to perform ALE of Al_2_O_3_, to understand how diketones bind to metal oxide surfaces, and to investigate the self-limiting mechanism of the diketone half-cycle. These first two research goals were achieved in Sections 3.1 and 3.2, 3.3, respectively. In this section, we discuss the possible etch products and present the hypothesized self-limiting mechanisms of both half-cycles.

The proposed etch product for this ALE chemistry is Al(hfac)_3_, similar to etch products proposed for continuous etching,^39,41,42^ and ALE.^7,28^ It may however be sterically difficult to form such a product at the surface.^46^ Another possibility is that AlH(hfac)_2_ or AlF(hfac)_2_ are formed as etch products. Formation of these species would be sterically easier as only two ligands need to bind around the Al atom. AlH(hfac)_2_ is a possible product considering that after the H_2_ plasma exposure there will likely be some H atoms bonded directly to surface Al atoms. Previous work has shown AlH_3_ to be highly volatile,^70^ and therefore substituting one of the hfac ligands from Al(hfac)_3_ with a H atom will likely also yield a volatile molecule. AlH_3_ formation does not appear to occur in this work as no continuous etching was observed when dosing only the H_2_ plasma half-cycle (Fig. 6(b)). As discussed earlier, there is a possibility that reactive fluorine-containing species such as CF_3_˙ or F˙ radicals are formed near the surface during the H_2_ plasma removal step, as a result of dissociation of hfac species in the H_2_ plasma. It is expected that recombination of F˙/CF_3_˙ with H˙ will occur to form HF/CHF_3_, due to the presence of H˙ radicals in the plasma. HF can fluorinate the Al_2_O_3_ surface to form AlF_3_, which has been shown to facilitate etching with Sn(acac)_2_ through the formation of volatile AlF_*x*_(acac)_*y*_ species.^7,71^ Similar reactions may occur in this work to form AlF(hfac)_2_ as the etch product. Further studies are required to identify the reaction products of the ALE chemistry.

From ALD literature there are two main mechanisms by which a reaction can saturate on a surface: having a limited number of reactive surface sites, or steric hinderance between the precursor ligands.^72,73^ In this study, a decrease in OH group absorbance was observed during the first 80 ms of Hhfac dosing in Fig. 9, which suggests that the OH groups are consumed during the initial Hhfac doses. Based on the DFT simulations this is likely due to the de-protonation of the Hhfac, which forms volatile H_2_O at the surface. Therefore, saturation of the Hhfac adsorption could occur once all the surface OH groups are consumed. However, with further Hhfac dosing there is still an increase in hfac absorbance in the wavenumber range 1900–1200 cm^−1^ (Fig. 9). From 80 ms to 500 ms of total Hhfac dosing time, the hfac absorbance increases without any change in the OH absorbance, suggesting that OH groups are not vital for hfac adsorption. Additionally, DFT analysis in the ESI† show that Hhfac binding to bare Al_2_O_3_ surfaces is energetically favorable (Fig. S7, ESI†). These results from experiments and simulations suggests that the presence of OH groups is not necessary for the adsorption of hfac ligands onto an Al_2_O_3_ surface, and therefore the mechanism of having limited reaction sites does not explain the self-limiting behavior.

**Fig. 14:**
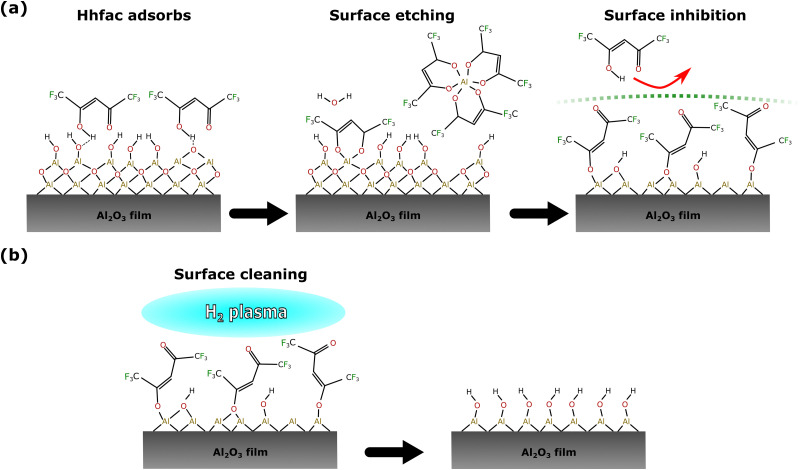
Proposed mechanism for ALE of metal oxides using diketone etch-inhibition and surface cleaning. (a) Adsorption of the Hhfac ligands onto the Al_2_O_3_ surface. Initial binding is in the chelate configuration, where both O atoms bind to the same surface atom, leading to the formation of volatile species. Over time, steric hinderance on the surface leads to the adsorption of more molecules in the monodentate and isolated chelate configurations, which eventually inhibit the etching. (b) The hfac species on the surface must then be cleaned away to reset the surface such that more etching can occur in the next ALE cycle. H_2_ plasma is used in this work to remove the surface inhibition layer.

The alternative pathway to a saturating surface reaction is that of steric hinderance, where reactive species are prevented from accessing the surface, thus preventing a continuous reaction. Evidence for such surface inhibition reactions during the diketone exposure can be seen in Fig. 10. Over consecutive Hhfac pulses the hfac absorbance increases and then saturates in the FTIR spectrum. For the saturated surface (after 500 ms total dose time) the hfac is primarily bound in monodentate configuration on the surface, with some contribution from the chelate configuration. The adsorbed hfac surface layer prevents additional Hhfac from reacting with the surface, inhibiting etching and thus explaining the self-limiting behavior of the first half-cycle as shown in Fig. 14(a). H_2_ plasma can then be used to remove the hfac surface layer as shown in Fig. 14(b), enabling etching in the next Hhfac half-cycle. Removal of Al atoms from the surface is thought to occur predominately during the initial doses of Hhfac, before the formation of the surface inhibition layer. In the first Hhfac doses, the adsorbed hfac species measured on the Al_2_O_3_ surface by FTIR are primarily in monodentate configuration. The initially high monodentate coverage is surprising considering that the simulations reported in this work and in AS-ALD literature suggest that the chelate configuration should be the most favorable surface species.^36^ This observation can be explained by a survivorship bias towards the monodentate configuration. On the initial Al_2_O_3_ surface, hfac can easily bind in the preferred chelate configuration and volatilize surface Al atoms, thereby leaving the surface and not appearing in the FTIR spectra.^7,27^ Some hfac species may bind in monodentate configuration as steric effects prevent them from transitioning into the chelate configuration (Fig. 12(c) and (d)), or they bind directly in monodentate through the less energetically favorable path (Fig. 13). As the surface becomes more crowded, steric hindrance will be more pronounced, and more Hhfac species will bind to the surface in monodentate configuration. Each Hhfac pulse adds more hfac in monodentate configuration to the surface, until the surface is saturated with monodentate bound hfac, which enables self-limitation of the Hhfac half-cycle. Additionally, some hfac species may bind in chelate configuration, but are surrounded by hfac ligands in monodentate configuration. Due to steric effects the monodentate ligands cannot transition into the chelate configuration. These isolated chelate species will not be able to form volatile Al-containing species as it is anticipated at least two chelate bound hfac ligands are required to make a volatile product. Thus, some chelate hfac ligands can contribute to the surface inhibition. Decomposition of the Hhfac into ketene structures may further contribute to the surface inhibition, accelerating the self-limiting behavior. These structures are not expected to be driving the mechanism as they only constituent a small part of the FTIR signal (Fig. 9). Taken together, it is hypothesized that during the Hhfac dose there is a competition between the etching and surface inhibition reaction.^33,44^

Once the etch-inhibition layer has formed, it can be cleaned away using a H_2_ plasma as demonstrated in Fig. 11. H radicals react with the hfac surface species to form volatile carbon-containing fragments, or reforming Hhfac which is volatile. Over repeated ALE cycles no buildup of hfac species was observed on the surface from FTIR measurements (Fig. 8), suggesting it is a good alternative to O_2_ plasma. By alternating between the etch/inhibition and surface cleaning half-cycles, the film thickness of Al_2_O_3_ can be controlled with sub-nm precision.

A small continuous etching component (0.003 ± 0.001 nm per step, Fig. 6(a)) is observed for this ALE chemistry. It is hypothesized that continuous etching occurs due to surface re-organization of the adsorbed hfac species. With continuous Hhfac dosing there is the possibility that some species may re-configure on the surface or desorb, which could open up space on the Al_2_O_3_ surface for new species to bind. If there is enough space at the newly available surface site, and the hfac ligand binds in chelate configuration to an Al atom that already had some chelate ligands bound to it, then it may be etched from the surface. As this relies on many factors lining up, the contribution to etching from this effect is minimal, as is reflected in the high synergy of this ALE process (98%). Further investigation is required to optimize Hhfac dosing conditions that lead to self-limiting etching and minimize the continuous etching. Based on previous literature and the work in this study, it is expected that high partial pressures of Hhfac will cause the reaction to favor a continuous etching regime,^39–42^ while repeated low exposure doses may favor the self-limiting regime.^34,44,45^

## 4. Conclusions

An ALE chemistry based on alternating doses of Hhfac and H_2_ plasma is developed, allowing for sub-nm control of film thickness with a high ALE synergy of 98%. The EPC was found to be strongly temperature-dependent, with higher temperatures leading to increased etch rates. High selectivity to SiO_2_ was demonstrated and shown to be due to the lack of adsorption of Hhfac on the SiO_2_ surface. Additionally, the EPC measured in this work was higher than the EPC of a comparable process using Hacac/O_2_ plasma.^34^ These observations highlight that Hhfac is a useful etchant for ALE, and that H_2_ plasma is an effective surface cleaning step which can be used as a substitute for O_2_ plasma. Etch-inhibition and surface cleaning ALE chemistries offer a good alternative to fluorination and ligand-exchange ALE, providing a route towards ALE with minimal surface contamination.

The mechanism of the Hhfac etching was explored in detail through FTIR and DFT analysis. It was observed that the self-limiting behavior of this process is dictated by the competitive adsorption of different diketone bonding configurations. Initially, species bind preferentially in the chelate configuration which leads to etching of the film. However, over multiple pulses the monodentate configurations build up on the Al_2_O_3_ surface, and sterically block adsorption of more Hhfac species on to the surface, thereby also inhibiting the etch reaction. A H_2_ plasma half-cycle can then be employed to remove the hfac species from the surface, exposing the Al_2_O_3_ surface and thus allowing for etching in the next Hhfac half-cycle. FTIR data shows no build-up of hfac species on the surface over multiple ALE cycles, further supporting H_2_ plasma as a suitable alternative to O_2_ plasma for surface cleaning.

For the development of other etch-inhibition and surface cleaning ALE processes, three required properties can be identified for the ALE etch/inhibitor molecule based on this work: Firstly, the molecule should be able to form volatile species with the film to be etched. Secondly, to enable self-limiting etching the molecule should have an alternative bonding configuration/surface reaction which leads to the formation of a stable surface inhibition layer. Finally, the inhibition should be easily removable, such that the surface is not poisoned over time. Diketones can be used as both etchants and surface inhibitors and can be easily removed by plasma or thermal reactants, making them an interesting group of molecules to explore for isotropic ALE.

## Data availability

The data that support the findings of this study are openly available in 4TU. ResearchData at https://data.4tu.nl/ (DOI: https://doi.org/10.4121/f2555371-5b4f-470a-b9ae-93ef5c8118ae).

## Conflicts of interest

There are no conflicts to declare.

## Supplementary Material

TC-013-D4TC03615H-s001
