# Supplementary material for: Investigation of the atomic layer etching mechanism for Al2O3 using hexafluoroacetylacetone and H2 plasma
Source: J Mater Chem C Mater. 2024 Nov 22;13(3):1345–58. doi: 10.1039/d4tc03615h (PMC11582961; doi:10.1039/d4tc03615h)
Supplement: TC-013-D4TC03615H-s001 [file TC-013-D4TC03615H-s001.pdf]

Supplementary Information (SI) for Journal of Materials Chemistry C.
This journal is © The Royal Society of Chemistry 2024

Supporting information

Investigation of the atomic layer etching mechanism for Al_2O_3 using hexafluoroacetylacetone and H_2 plasma

*Nicholas J. Chittock[1,2], Joost F.W. Maas[1], Ilker Tezsevin[1], Marc J.M. Merkx[1], Harm C.M. Knoops[1,2], Wilhelmus M.M. (Erwin) Kessels[1] and Adriaan J.M. Mackus[1]**

AUTHOR ADDRESS

1. Department of Applied Physics, Eindhoven University of Technology, P.O. Box 513, 5600 MB Eindhoven, The Netherlands

2. Oxford Instruments Plasma Technology, North End, Bristol BS49 4AP, United Kingdom

DIKETONE CONTINUOUS ETCH COMPONENT

A small continuous etching component is observed when dosing only the Hhfac precursor. Figure 4 of the main text shows the synergy plot for this ALE reaction, with a calculated synergy of 98 %. The deviation in synergy from 100 % is due to the small continuous etch component of 0.003 nm/cycle observed for the Hhfac dose in Figure 6a. The continuous etch component can be seen more clearly in Figure S1. Previously Hhfac has been used as a continuous etchant when dosed at high partial pressures.[1,2] In the first pulse in Figure S1 there is adsorption of Hhfac denoted

by the increase in film thickness. After the first pulse, as more Hhfac is dosed, the film thickness starts to decrease. By dosing more Hhfac there can be re-organization of hfac ligands on the surface leading to a small continuous etch component as discussed in Section 3.2. The high synergy of the ALE process (98 %) shows that the continuous etch component is very small and that the ALE process offers a high level of control.

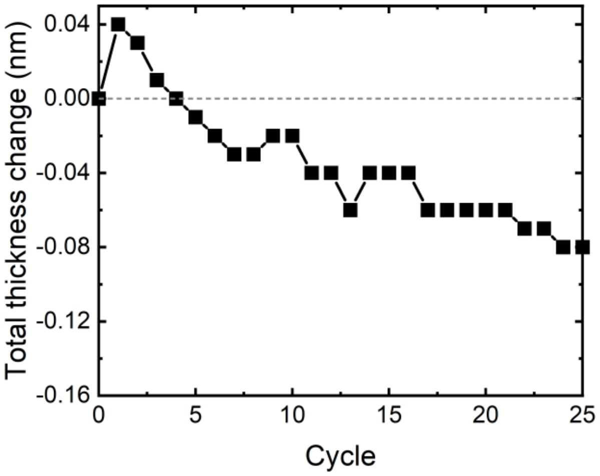

Figure S1: Al_2O_3 film thickness change as a function of Hhfac exposure at 350 ˚C. The initial dose shows surface adsorption of the diketone species, but upon further dosing a continuous etch component is observed. A straight line fit of the etching region yields an EPC of 0.003 nm/cycle.

TEMPERATURE DEPENDENCE OF ETCH RATE

Previously it has been reported that using high temperatures can increase the etch rate when etching with diketone species.[1,3,4] In this work a higher substrate temperature also leads to a higher EPC for Al_2O_3, as shown in Figure S2. EPC values of 0.04 ± 0.01 nm, 0.08 ± 0.01 nm and 0.16 ± 0.02 nm at 250 ˚C, 300 ˚C and 350 ˚C, respectively, are measured. The EPC recorded at 350 ˚C is in agreement with the EPC measured in the saturation curves at 350 ˚C (see Figure 5). Going to

temperatures above 350 ˚C may result in lower etch rates as decomposition of the Hhfac will occur.[1] Previously, for continuous etching of ZnO with Hhfac, a maximum etch rate was observed at 350 ˚C substrate temperature, however this coincided with a high level of C and F contamination of 67 % and 4 %, respectively.[1]

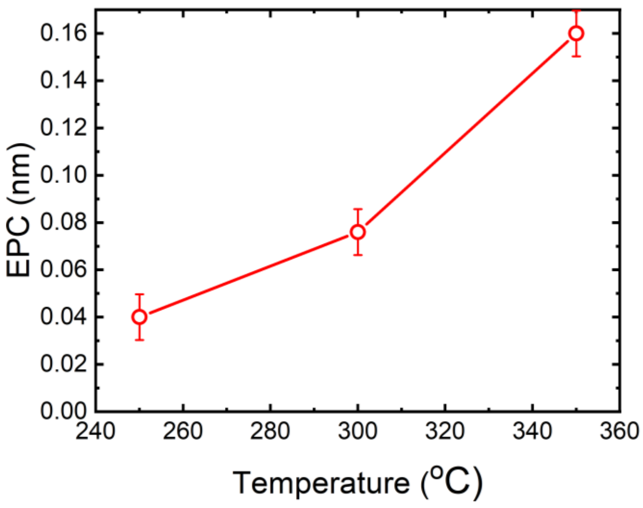

Figure S2. EPC as a function of temperature for the ALE process consisting of 15x50 ms Hhfac pulses and 25 s H_2 plasma per cycle.

ROUGHNESS REDUCTION

A reduction in roughness is observed post ALE as can be seen in Figure S3, with the RMS roughness decreased from 0.50 ± 0.07 nm to 0.24 ± 0.07 nm. In the current study, the roughness value approaches that of the pristine Si substrate underneath (0.20 ± 0.05 nm). Based on previous ALE smoothing work this can be quantified as a smoothing rate of 0.10 ± 0.03 nm/nm, which is significantly higher than that observed for the SF_6 plasma/TMA ALE chemistry (0.0098 ± 0.002 nm/nm).[5] Literature reports on ALE with diketones have shown that the roughness is either unchanged or reduced post ALE.[6–8]

A possible hypothesis as to why the Hhfac/H_2 plasma ALE process leads to smoothing is the uniform front propagation + curvature dependent (UFP+CD) model proposed by Gerritsen & Chittock *et al.*.[5] The model proposes that ALE smooths the surface by the etch front uniformly propagating into the material, with slightly enhanced etching at convex regions (peaks) and reduced etching in concave regions (valleys) of the film. The rate of surface propagation, *F*, in the model is given by the equation $F=1-\varepsilon K$, where K is the local curvature of the surface.[5] The coefficient, ε, is then determined from fitting experimental data, and describes how heavily the local curvature influences the rate of smoothing. Over the ALE cycles the slight differences in EPC act to smoothen the surface as nanoscale peaks and valleys are levelled out.

The UFP+CD model can be used to fit the Hhfac/H_2 plasma roughness reduction with reasonable accuracy if a large coefficient ($\varepsilon = 9\cdot10^{-9}$ m) is used, as shown in Figure S3. For comparison, the coefficient is $\varepsilon = 1.5\cdot10^{-9}$ m for Al_2O_3 (and $0.05\cdot10^{-9}$ m for GaN) when using an SF_6 plasma/TMA ALE chemistry. This may highlight that the local curvature of the surface plays an important role in the smoothing mechanism for the Hhfac/H_2 plasma ALE chemistry. The dependence on local curvature could be attributed to the preferential etching of adatoms and protrusions from the surface, and to the difficulty of etching concave regions of the film. Previous DFT simulations have shown that removal of adatoms from a surface with diketones is more energetically favorable than removal of a terrace atom.[6,7,9] Additionally, the steric hinderance of adsorbing sufficient diketone species around an atom to make a volatile product is less significant if the atom protrudes from the substrate surface. The inverse occurs in concave regions, where adsorption is more sterically hindered. Additional experiments are required to elucidate the exact nature of the smoothing mechanism for this ALE chemistry.

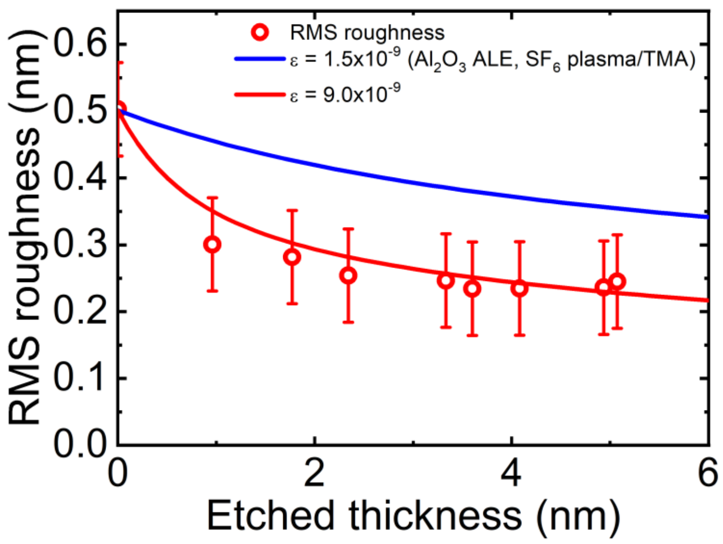

Figure S3. Evolution of film roughness as a function of ALE etched thickness at 350 ˚C. The RMS roughness decreases rapidly and then saturates around 0.24 ± 0.07 nm, similar to the RMS roughness of the underlying Si substrate. The two lines are fit using the uniform front propagation + curvature dependence model from Gerritsen & Chittock *et al.* with different values for the coefficient, ε, and the same starting surface roughness.[5]

SIMULATED BOND VIBRATIONAL FREQUENCIES

To aid the infrared peak identification the bond vibrational frequencies for the different hfac bonding configurations on an Al_2O_3 surface were simulated. The optimized geometries shown in Figure 11 were used as the inputs. Vibrational frequencies were calculated in VASP as outlined in the main text. Vibrations attributed to the different bonding configurations are listed in Table S1.

Table S1. The main peaks identified for the different hfac bonding configurations. In bold are the wavenumbers used to identify the monodentate and chelate peaks.

**Binding configuration**		**Wavenumber (cm^{-1})**											
**Etch**	**Chelate**	1629	1613	**1569**	**1558**	**1557**	1476	1473	1323	1319	1216		

**Non-etch**	**Monodentate**	1696	**1655**	1649	1641	1623	1614	1606	1516	1477	1432	1355	1266

TOTAL HHFAC ABSORBANCE SPECTRA

Figure S4 shows the absorbance spectra for an Al_2O_3 surface with adsorbed hfac species referenced to the as-deposited Al_2O_3. The integrated area of the spectrum is used to plot Figure 6d of the main text. The change in absorbance after each Hhfac pulse is decreasing, suggesting that fewer hfac ligands are being added to the surface with each pulse. Eventually there is minimal change in the spectrum when dosing more Hhfac, indicating that the surface has become saturated with hfac ligands.

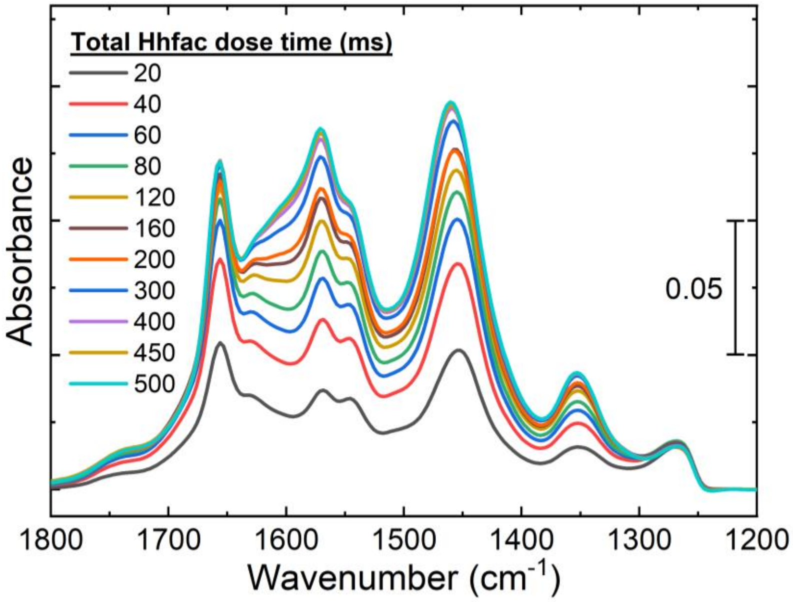

Figure S4. Absorbance spectra for an Al_2O_3 surface exposed to Hhfac pulses, referenced to the as-deposited Al_2O_3 film. For total Hhfac dose times longer than 400 ms there is negligible change in the spectra highlighting that the surface is saturated.

UTILIZATION OF O2 PLASMA FOR SURFACE CLEANING

Earlier ALE work used O_2 plasma as the surface cleaning step. However, area-selective ALD studies have shown that H_2 plasma is a more effective cleaning step.[3,10] Therefore, in this work H_2 plasma is used to develop the ALE process. However, it is still of interest to see if the O_2 plasma could be used for removal of hfac ligands from an Al_2O_3 surface.

A planar Al substrate coated in ALD Al_2O_3 is exposed to a 250 ms Hhfac exposure, resulting in the spectra shown in Figure S6. When subsequently exposing the hfac-functionalized surface to a 20 s 100 W O_2 plasma, there is clear removal of the adsorbed hfac ligands. Together with previous literature, Figure S5 suggests that an O_2 plasma would be a viable alternative surface cleaning half-cycle for this ALE chemistry.[3]

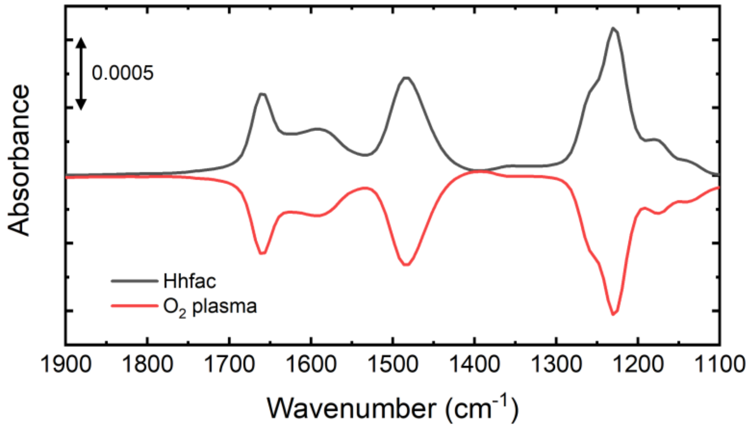

Figure S5. RAIRS spectra taken in reflection mode for an Al_2O_3 film exposed to a 250 ms Hhfac exposure, referenced to the as-deposited Al_2O_3 film. The spectra are shown in the wavenumber range (1900-1100 cm^{-1}). After Hhfac exposure (black line), there are clear peaks indicating adsorption of hfac ligands. After subsequent exposure to an O_2 plasma (red line, referenced to the 250 ms Hhfac spectra) there are negative peaks in the spectra. The spectrum after O_2 plasma is a mirror of the Hhfac adsorption, suggesting that all the added hfac ligands are removed.

DFT ANALYSIS

The monodentate configuration shown in Figure 12b is hypothesized to contribute solely to the etch inhibition layer and cannot transition into the chelate configuration. As the hydroxyl group of the hfac is pointing away from the surface, a rotation of the carbon backbone would be required for the molecule to bind in the chelate configuration. To investigate whether the Hhfac molecule can rotate on the surface, gas phase studies were performed looking into the energy barrier for the backbone rotation. Using Jaguar software with B3LYP-D3 and 6-31 G** basis set, the energy profile in Figure S6 was obtained. The graph shows that to transition into a configuration where both O atoms would be directed at the surface, there is a ~1 eV energy barrier. Due to this energy barrier, it is unlikely that a molecule which initially binds in the monodentate configuration in Figure 13b will transition in to a chelate configuration. Therefore, the configuration shown in Figure 13b is expected to only contribute to etch inhibition.

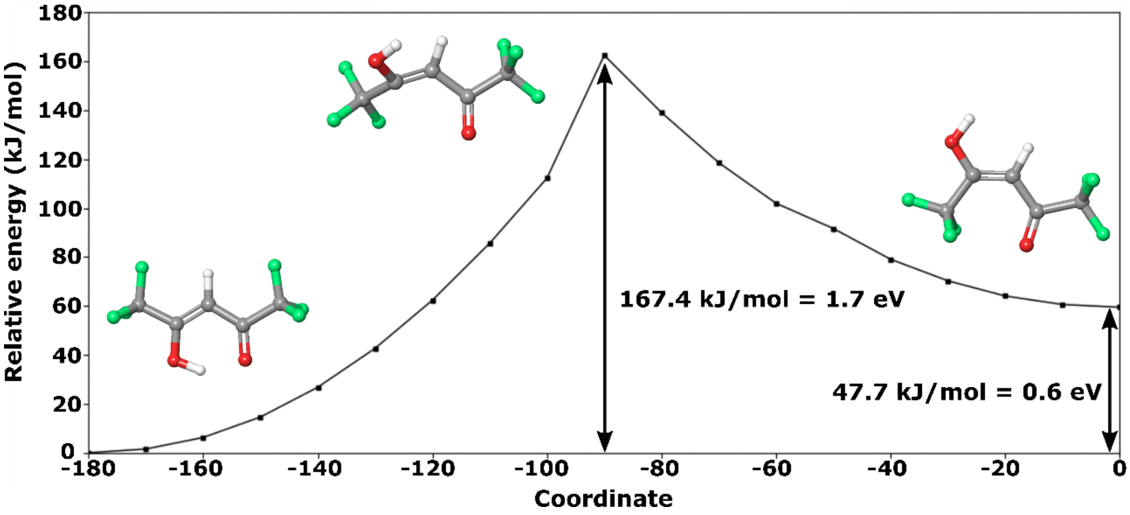

Figure S6. Energy barrier for rotation of the C=C bond in the carbon backbone of a Hhfac molecule in gas phase.

Adsorption of Hhfac was also investigated on a bare Al_2O_3 surface to investigate whether OH groups are a necessary reaction site for adsorption. The same level of theory as in the main DFT

studies was used for these analysis. As shown in Figure S7 the physisorption of Hhfac is more energetically favorable on the bare Al_2O_3 (ΔE_{ads}=-0.93 eV) than on the OH-terminated Al_2O_3 surface (ΔE_{ads}=-0.60 eV). Furthermore, the monodentate and chelate configurations also have a lower energy configuration on the bare Al_2O_3 compared to the OH-terminated. A difference between the two surfaces is that for the bare Al_2O_3 the H from the Hhfac molecule is donated to a surface O atom rather than to an OH group, forming OH at the surface. Similar to the OH-terminated surface, the lowest energy configuration is the chelate configuration, supporting the hypothesis that *if sterically possible* the Hhfac will bind in chelate configuration. These simulations highlight that Hhfac can strongly bind to the Al_2O_3 surface, and that OH groups are not required to mediate adsorption. Thus, availability of surface OH groups is likely not a cause of self-limiting etching.

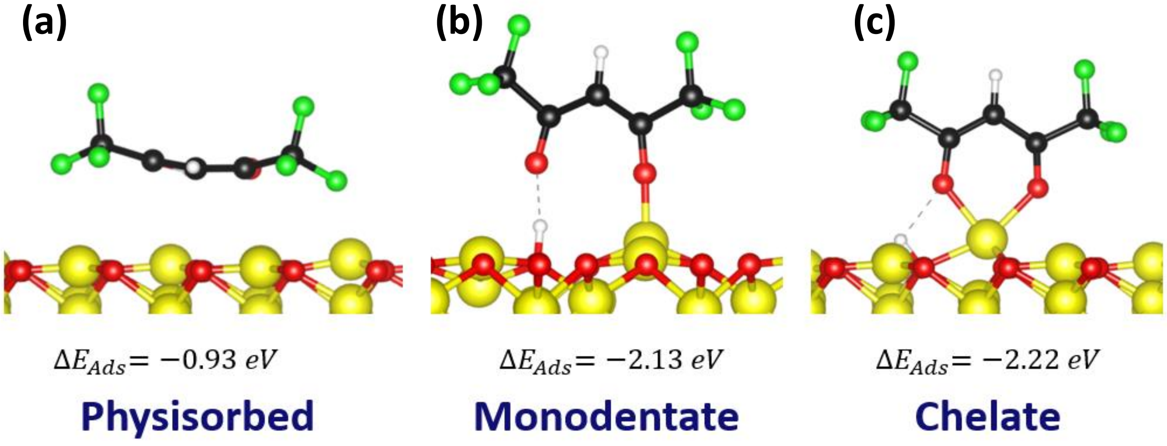

Figure S7. Adsorption energy for the different binding configurations on the bare-Al_2O_3 surface computed in VASP as described in the main text.

REFERENCES

(1) Droes, S. R.; Kodas, T. T.; Hampden-Smith, M. J. Etching of ZnO Films with Hexafluoroacetylacetone. *Advanced Materials* **1998**, *10* (14), 1129–1133.

https://doi.org/10.1002/(SICI)1521-4095(199810)10:14<1129::AID-ADMA1129>3.3.CO;2-9.

(2) Droes, S. R.; Jain, A.; Kodas, T. T.; Hampden-Smith, M. J.; Muenchausen, R. Utilization of SS-Diketones for Etching and Cleaning of Metal Oxide and Sulfide Thin Films. *MRS Proceedings* **1993**, *300* (8.5.2017), 471. https://doi.org/10.1557/PROC-300-471.

(3) Mameli, A.; Verheijen, M. A.; Mackus, A. J. M.; Kessels, W. M. M.; Roozeboom, F. Isotropic Atomic Layer Etching of ZnO Using Acetylacetone and O 2 Plasma. *ACS Appl Mater Interfaces* **2018**, *10* (44), 38588–38595. https://doi.org/10.1021/acsami.8b12767.

(4) Gertsch, J. C.; Cano, A. M.; Bright, V. M.; George, S. M. SF 4 as the Fluorination Reactant for Al 2 O 3 and VO 2 Thermal Atomic Layer Etching. *Chemistry of Materials* **2019**, *31* (10), 3624–3635. https://doi.org/10.1021/acs.chemmater.8b05294.

(5) Gerritsen, S. H.; Chittock, N. J.; Vandalon, V.; Verheijen, M. A.; Knoops, H. C. M.; Kessels, W. M. M.; Mackus, A. J. M. Surface Smoothing by Atomic Layer Deposition and Etching for the Fabrication of Nanodevices. *ACS Appl Nano Mater* **2022**, *5* (12), 18116–18126. https://doi.org/10.1021/acsanm.2c04025.

(6) Konh, M.; Janotti, A.; Teplyakov, A. Molecular Mechanism of Thermal Dry Etching of Iron in a Two-Step Atomic Layer Etching Process: Chlorination Followed by Exposure to Acetylacetone. *The Journal of Physical Chemistry C* **2021**, *125* (13), 7142–7154. https://doi.org/10.1021/acs.jpcc.0c10556.

(7) Konh, M.; He, C.; Lin, X.; Guo, X.; Pallem, V.; Opila, R. L.; Teplyakov, A. V.; Wang, Z.; Yuan, B. Molecular Mechanisms of Atomic Layer Etching of Cobalt with Sequential Exposure to Molecular Chlorine and Diketones. *Journal of Vacuum Science & Technology A: Vacuum, Surfaces, and Films* **2019**, *37* (2), 021004. https://doi.org/10.1116/1.5082187.

(8) Konh, M.; Wang, Y.; Chen, H.; Bhatt, S.; Xiao, J. Q.; Teplyakov, A. V. Selectivity in Atomically Precise Etching: Thermal Atomic Layer Etching of a CoFeB Alloy and Its Protection by MgO. *Appl Surf Sci* **2022**, *575* (October 2021), 151751. https://doi.org/10.1016/j.apsusc.2021.151751.

(9) Basher, A. H.; Krstić, M.; Fink, K.; Ito, T.; Karahashi, K.; Wenzel, W.; Hamaguchi, S. Formation and Desorption of Nickel Hexafluoroacetylacetonate Ni(Hfac)2 on a Nickel Oxide Surface in Atomic Layer Etching Processes. *Journal of Vacuum Science & Technology A: Vacuum, Surfaces, and Films* **2020**, *38* (5), 052602. https://doi.org/10.1116/6.0000293.

(10) Merkx, M. J. M.; Sandoval, T. E.; Hausmann, D. M.; Kessels, W. M. M.; Mackus, A. J. M. Mechanism of Precursor Blocking by Acetylacetone Inhibitor Molecules during Area-Selective Atomic Layer Deposition of SiO 2. *Chemistry of Materials* **2020**, *32* (8), 3335–3345. https://doi.org/10.1021/acs.chemmater.9b02992.
